# Implications of the Use of Eukaryotic Translation Initiation Factor 5A (eIF5A) for Prognosis and Treatment of Hepatocellular Carcinoma

**DOI:** 10.1155/2012/760928

**Published:** 2012-09-18

**Authors:** Felix H. Shek, Sarwat Fatima, Nikki P. Lee

**Affiliations:** Department of Surgery, The University of Hong Kong, Pokfulam, Hong Kong

## Abstract

Hepatocellular carcinoma (HCC) is a primary liver malignancy and accounts for most of the total liver cancer cases. Lack of treatment options and late diagnosis contribute to high mortality rate of HCC. In eukaryotes, translation of messenger RNA (mRNA) to protein is a key process in protein biosynthesis in which initiation of translation involves interaction of different eukaryotic translation initiation factors (eIFs), ribosome subunits and mRNAs. Eukaryotic translation initiation factor 5A (eIF5A) is one of the eIFs involved in translation initiation and eIF5A2, one of its isoforms, is upregulated in various cancers including HCC as a result of chromosomal instability, where it resides. In HCC, eIF5A2 expression is associated with adverse prognosis such as presence of tumor metastasis and venous infiltration. Based on eIF5A2 functional studies, suppressing eIF5A2 expression by short interfering RNA alleviates the tumorigenic properties of HCC cells *in vitro* while ectopic expression of eIF5A2 enhances the aggressiveness of HCC cells *in vivo* and *in vitro* by inducing epithelial-mesenchymal transition. In conclusion, eIF5A2 is a potential prognostic marker as well as a therapeutic target for HCC.

## 1. Clinical Situation of Liver Cancer

Liver cancer is one of the most prevalent and lethal malignancies worldwide. It is the second most frequent cause of cancer deaths and the fifth most common diagnosed cancer in men. Liver cancer is prevalent in Southeast Asia and Africa, but the incidence rates are also on the rise in America and Europe. The number of incident cases worldwide is over 740,000 per year, and the number of mortality cases is similar to the incident cases. Hepatocellular carcinoma (HCC) represents the major subtype of primary liver cancer, accounting for 70–85% of total liver cancer cases [1]. High-risk population of HCC includes cirrhosis patients and hepatitis B or C virus carriers. Other risk factors include aflatoxin intake, obesity, and alcohol abuse [2]. The high mortality rate of HCC is a result of lack of treatment options and late diagnosis. A few commonly used diagnostic methods for HCC include ultrasonography and detection of alpha-fetoprotein in the serum. Early stage HCC is often asymptomatic, and most HCCs are diagnosed at an advanced stage where treatment options are limited. Currently, curative treatment options for patients diagnosed with early stage HCC include surgical resection of tumor and liver transplantation. However, surgical resection results in a high rate of postsurgical recurrence, and it is not suitable for patients with impaired liver functions. While for liver transplantation, shortage of liver grafts remains a major challenge. Another treatment option for HCC patients is transarterial chemoembolization (TACE). TACE is a minimally invasive procedure where blood flow to the tumor is blocked and chemotherapeutic agents are administered directly to the tumor. However, patients with impaired liver function are not suitable for TACE treatment as it may lead to severe complications due to liver failure [2]. Targeted therapy offers an alternative for advanced stage HCC patients who are not suitable for curative treatments or TACE. Sorafenib, the only FDA-approved targeted therapy for the treatment of advanced HCC, is a multikinase inhibitor targeting several different kinases including vascular endothelial growth factor receptor and platelet-derived growth factor receptor. It is also the only systematic agent found to increase the survival time of patients by about 3 months. However, in addition to its restricted use in advanced HCC, it cannot be administered to HCC patients with severe cardiovascular disease and portal hypertension [3, 4]. Based on the current clinical situation of HCC, lack of effective treatment options is a major factor leading to the high mortality rate of HCC. Thus, it is necessary to develop new treatments that can be used for HCC patients under a wide range of conditions. Based on our previous study and other studies, it is proposed that eukaryotic translation initiation factors (eIFs) constitute a potential class of therapeutic targets for treatment of various cancers, and this paper will focus on discussing the implications of using eukaryotic translation initiation factor 5A (eIF5A) as a prognostic marker and treatment target in HCC.

## 2. Protein Synthesis and eIFs

In eukaryotes, translation of messenger RNA (mRNA) to a polypeptide is a key process in protein synthesis. It consists of three main steps: (1) initiation of translation, (2) elongation of polypeptide chain, and (3) termination of translation. In brief, the initiation step involves the assembly of different ribosomal subunits, initiation factors, and mRNA to form an 80S ribosomal complex. Transfer RNAs (tRNAs), carrying specific amino acids, recognize the codon of mRNA and bind to the 80S ribosomal complex to start the initiation step. As the ribosomal complex moves along the mRNA, different tRNAs recognize their corresponding codon and bring amino acids to the ribosomal complex to form a polypeptide chain. The translation process terminates when the ribosomal complex encounters a termination signal. This termination codon cannot be recognized by tRNAs. The polypeptide chain is then released from the ribosomal complex and proceeds to posttranslational modification while the 80S ribosomal complex disassembles and recycles again [5–9].

The eIF family represents a group of proteins that are involved in the initiation step of protein translation. Each member plays a unique role in the initiation process by interacting with ribosomal subunits and mRNAs to form an elongation competent complex [6]. There are at least ten different eIFs taking part in the initiation step of translation, which involves eIF interaction with mRNA, 40S and 60S ribosomal subunits to form an 80S ribosomal complex. In brief, eIF4A, eIF4B, eIF4E, and eIF4G activate mRNA while eIF1, eIF1A, eIF2, eIF3, and eIF5 interact with 40S and 60S ribosomal subunits. The activated mRNA subsequently binds to the 40S and 60S subunits to form the 80S ribosomal complex, which initiates translation [6, 8, 9]. The following sections will discuss the protein synthesis pathway and in particular the protein translation control process in cancer development.

## 3. Deregulation of Protein Synthesis Pathway Frequently Observed in Cancers

Total protein levels in cells are regulated by two cellular mechanisms, the ubiquitin system and the protein synthesis mechanism. It is well known that the ubiquitin-mediated degradation of protein is important for regulating different cellular activities such as transcription, signal transduction and cell-cycle progression [10–13], while protein synthesis is vital for protein generation and determination of cellular phenotype. Under normal conditions, the protein synthesis pathway is well regulated to prevent overproduction of proteins. However, aberrant activation of this pathway, such as those associated with mammalian target of rapamycin (mTOR), is observed in various cancers, leading to uncontrolled protein synthesis and the subsequent transformation of normal cells to more aggressive cancerous cells [14, 15]. Dysregulation of cellular signaling pathways is one of the hallmarks of cancer [16, 17]. For example, the mitogen-activated protein kinase (MAPK) [18] and the phosphoinositide 3-kinase (PI3 K)-Akt [19, 20] pathways are constitutively activated in various cancers. These pathways regulate cellular activities such as proliferation and differentiation but accumulative mutations impair the regulatory mechanism of these pathways that render cells with growth and survival advantages. Dysregulation of the protein synthesis pathway, similar to other deregulated signaling pathways, sometimes is a result of accumulative mutations in a cell. For example, deregulation can be caused by aberrant expression of eIFs together with aberrant activation of mTOR signaling pathway [7]. It has been suggested that restoring the aberrant activated pathway may inhibit cell transformation. However, further research is warranted to support this idea. It is found that there are different eIFs aberrantly expressed in liver tumors (Table 1), the content below will focus on one eIF family member, eIF5A, which is a unique member of the protein synthesis pathway and has been reported to have an oncogenic role in cancer development.

## 4. eIF5A and Its Isoforms

eIF5A is a small molecular-sized protein classified in the eIF family. It is conserved in all organisms from bacteria to humans, except in eubacteria. eIF5A mainly functions as an elongation factor in mRNA translation by facilitating the formation of the first peptide bond during the translation initiation step. eIF5A also serves as a shuttle protein regulating the nucleus-cytoplasmic transport of mRNAs in cells [24–26]. eIF5A is the only protein in eukaryotes to contain the amino acid residue hypusine. Hypusine, [N^*ε*^-(4-amino-2-hydroxybutyl)lysine], is a polyamine-derived amino acid which is formed by the post-translational modification of lysine in a process known as hypusination. This is a two-step enzymatic reaction which activates eIF5A. In the first step, the deoxyhypusine synthase (DHS) catalyzes the transfer of the 4-aminobutyl moiety of spermidine to the lysine residue (Lys50) of eIF5A precursor (inactive form) to form an intermediate, deoxyhypusine residue. This intermediate is sequentially hydroxylated by the enzyme deoxyhypusine hydroxylase (DOHH) to form a hypusine residue to complete the process [25, 27]. The eIF5A precursor is activated by hypusination and converted to a functional mature eIF5A. eIF5A is important in translation initiation since disruption of hypusination process by the DHS inhibitor, N^1^-guanyl-1,7-diaminoheptane (GC7), has been shown to inhibit growth of endothelial cells [28]. 

In humans, two isoforms of eIF5A have been identified sharing 80% cDNA sequence and 94% protein similarity [29, 30]. eIF5A1 is predominantly expressed in most mammalian cells, whereas eIF5A2 is differentially expressed in specific tissues such as testis and brain [30, 31]. The genes encoding isoforms eIF5A1 and eIF5A2 are located on different chromosomes, suggesting their different functional roles. The eIF5A1 gene resides on chromosome 17p12-p13 whereas eIF5A2 gene resides on chromosome 3q25-q27, a region where amplification is observed in different human malignancies [32–36]. Therefore, eIF5A2 has been proposed as an oncogene which could contribute to carcinogenesis and tumor progression, suggesting further research on the roles of eIF5A2 in HCC. 

## 5. Aberrant Expression of eIF5A Isoforms in HCC and Other Cancers

Various approaches have been used to study the genomic and proteomic profile of cancer cells. Differential expression of the two eIF5A isoforms has been observed in different cancers (Table 2). It is suggested that up-regulation of eIF5A expression contributes to proliferation of cancer cells most likely by constitutive activation of the protein synthesis pathway. In HCC, eIF5A2 is aberrantly expressed at mRNA and protein levels, whereas no change in expression level of eIF5A1 has been observed. In addition, tissues with up-regulation of eIF5A2 also show high levels of DHS and DOHH at the transcript level [37], indicating constitutive activation of eIF5A2 synthesis and hypusination resulting in increased protein synthesis and cell growth in HCC. Utilizing comparative genomic hybridization and copy number variation analysis, chromosome 3q is amplified in HCC [38], as well as in several cancers including pancreatic [36, 39], esophageal [40, 41], prostate [42], lung [32, 43], gastric [44, 45], ovarian [33, 46] and colorectal [30, 47] cancer. Thus, the aberrant expression of eIF5A2, also residing on chromosome 3q, is more frequently observed than that of eIF5A1 and this may at least partially explain for the aberrant expression of eIF5A2 in HCC. In addition, chromosome 3q amplification has also been correlated to HCC recurrence [48]. Thus, the role of eIF5A2 in HCC recurrence warrants further research.

Aberrant expression of the two eIF5A isoforms is reported in various cancers other than HCC. It is demonstrated that there is an up-regulation of eIF5A1 in colorectal adenoma by comparing the proteomic profiles of colorectal adenoma and normal mucosa using 2-dimensional electrophoresis proteomic profiling [49]. In ovarian cancer, eIF5A2 overexpression promotes tumorigenesis both *in vitro* and *in vivo* and is also positively correlated to an advanced stage of the disease [46]. In a recent study of non-small cell lung cancer (NSCLC), He et al. reported up-regulation of eIF5A2 in about 40% of tumor tissues by immunohistochemistry. This aberrant expression of eIF5A2 was positively correlated with advanced tumor stage and also indicated poor prognosis for early stage NSCLC patients. Furthermore, fluorescence *in situ *hybridization demonstrated amplification of eIF5A2 gene in NSCLC tumors. Therefore, up-regulation of eIF5A2 may result from genetic instability of chromosome 3q and may serve as a prognostic marker for early stage NSCLC [51].

Based on the above studies, we postulated that the differential expression and function of the two eIF5A isoforms are contributed partially by genetic instability of cancer cells. eIF5A1 gene resides on a genetically stable chromosome whose aberrant expression in cancers is not frequent. In addition, eIF5A1 is expressed in most mammalian cells implying that the function of eIF5A1 is to maintain basal level of mRNA translation in cells. While for eIF5A2, the gene resides on a genetically unstable chromosome whose amplification is frequently observed in various cancers. Chromosome 3q amplification leads to up-regulation of eIF5A2 in cancers, disturbing the well-regulated translational level maintained by eIF5A1 and leading to aberrant activation of protein synthesis pathway; as the result, normal cells are transformed to more aggressive cancerous cells. 

## 6. Prognostic Implications of eIF5A Isoforms in HCC

This section will describe and discuss the correlation of eIF5A isoforms and clinical characteristics of HCC. Using cDNA microarray analysis, Lee et al. found an association between high levels of eIF5A1 and eIF5A2 with increased number of tumor nodules and presence of venous infiltration, respectively, in patients [37]. In consistence with these findings, another study using quantitative polymerase chain reaction reported a reciprocal association between up-regulation of eIF5A2 with tumor metastasis and encapsulation [50]. These results suggest the differential roles of eIF5A1 and eIF5A2 in HCC development. Aberrant mRNA expression of eIF5A1 promotes the formation of tumor nodules, whereas up-regulation of eIF5A2 mRNA indicates adverse prognosis in HCC, as its expression may predict metastasis and tumor invasion. However, as a cytoplasmic protein, eIF5A2 expression can only be assessed at the mRNA level or by intracellular localization. It would also be clinically significant, for ease of diagnosis and minimal invasion, if a secretory form of eIF5A2 can be identified in patient serum or blood, but until now, this has not been reported in any malignancy. Although eIF5A1 mRNA expression is similar in liver tumor and non-tumor tissues, its expression is positively correlated to the number of tumor nodules in HCC. Thus, a combined analysis of eIF5A1 with other HCC tumor growth markers may improve its prognostic potential.

## 7. Therapeutic Potential of Targeting eIF5A2 in HCC

Given their association to different clinical characteristics of HCC, it is of interest to study the therapeutic potential of eIF5A isoforms for HCC treatment. Several studies have investigated the role of eIF5A2 as a potential therapeutic target for HCC. Loss-of-function study by Lee et al. demonstrated inhibition of cell growth and reduction of cell migration in HCC cells upon suppression of eIF5A2 using short interfering RNA (siRNA)[37]. Further demonstrating this, Tang et al. reported by a gain-of-function study in which overexpression of eIF5A2 in HCC cells promotes cell migration *in vitro* and tumorigenicity *in vivo*. Additionally, ectopic expression of eIF5A2 induced epithelial-mesenchymal transition, characterized by the downregulation of epithelial markers, *β*-catenin and E-cadherin, and up-regulation of mesenchymal markers including vimentin and N-cadherin. Moreover, overexpression of eIF5A2 also induced Rho/Rac GTPases activity to facilitate reorganization of actin cytoskeleton and disruption of adherent junctions [50]. Together these studies demonstrate the potential of eIF5A2 siRNA treatment as an adjuvant therapy for HCC patients. Antibody treatment offers an alternative to targeting eIF5A2 but currently there is no report of eIF5A2 antibody therapy in HCC. Other than targeting eIF5A2 alone, the effectiveness of combined therapy has also been investigated. Combined treatment of eIF5A2 siRNA and GC7, an inhibitor of DHS, on HCC cells resulted in a synergistic inhibition of cell migration [50]. As described in the previous section, inhibition of DHS activity by GC7 disrupts the hypusination process, abolishing eIF5A2 activation and leading to eIF5A precursor accumulation in cells [28, 31]. Further studies are warranted to investigate the effectiveness of combined treatment *in vivo *for the development of eIF5A2-targeted therapy in HCC.

## 8. Summary

In summary, eIF5A, an indispensable member of the translation initiation process, is found to be aberrantly expressed in different malignancies including HCC, ovarian cancer, and lung cancer. One of its isoforms, eIF5A2, is overexpressed in HCC tissues, and this up-regulation may be a result of chromosome 3q amplification where the eIF5A2 gene resides. Clinical studies have demonstrated a correlation between up-regulation of eIF5A2 level with tumor metastasis and venous infiltration. Therefore, eIF5A2 has been proposed as an indicator of tumor invasiveness in HCC. In addition, targeting eIF5A2 by siRNA and combined treatment with GC7 effectively reduces the migration ability of tumor cells, suggesting that targeting eIF5A2 and hypusination could be a potential treatment for HCC.

## Figures and Tables

**Table 1:** Differential expression of different eIFs in liver tumors.

Eukaryotic translation initiation factor (eIF)	Expression level	Clinical correlation with high level of eIF	References
eIF3S3	Induction	Large tumor size, presence of HBV infection	[21]
eIF4A1	Induction	NS	[22]
eIF4E	Induction	Increased tumor invasiveness	[22, 23]

NS: not studied.

**Table 2:** Aberrant expression of eIF5A1 and eIF5A2 in different human cancers.

Malignancy	Expression in tumor		Chromosome 3q	Clinical correlation with	References
	eIF5A1	eIF5A2	amplification	high eIF5A level
Colorectal cancer	Induction	Induction	Presence	NS	[30, 47, 49]
Esophageal cancer	NS	Induction	Presence	NS	[40, 41]
Gastric cancer	NS	NS	Presence	NS	[44, 45]
Liver cancer	Unchanged	Induction	Presence	Increased number of tumor nodules, presence of tumor venous infiltration	[37, 38, 48, 50]
Lung cancer	NS	Induction	Presence	Advanced tumor stage, poor survival of patients	[32, 43, 51]
Ovarian cancer	NS	Induction	Presence	Advanced tumor stage	[33, 46]
Pancreatic cancer	NS	Induction	Presence	NS	[36, 39, 52]
Prostate cancer	NS	NS	Presence	NS	[42]

NS: not studied.
